# Transcriptome network data in larval zebrafish (*Danio rerio)* following exposure to the phenylpyrazole fipronil

**DOI:** 10.1016/j.dib.2020.106413

**Published:** 2020-10-16

**Authors:** Ashley Eadie, Isabel Cristina Vásquez, Xuefang Liang, Xiaohong Wang, Christopher L. Souders, Jana El Chehouri, Rohit Hoskote, April Feswick, Andrew M. Cowie, Jennifer R. Loughery, Christopher J. Martyniuk

**Affiliations:** a Department of Biology, University of New Brunswick, Saint John, New Brunswick, E2L 4L5, Canada; b Departamento de Ciencias Fisiológicas, Facultad de Medicina, Pontificia Universidad Javeriana, Bogotá D.C., Colombia; c School of Ecology and Environment, Inner Mongolia University, Hohhot, Inner Mongolia, 010021, People's Republic of China; d Department of Physiological Sciences and Center for Environmental and Human Toxicology, University of Florida Genetics Institute, Interdisciplinary Program in Biomedical Sciences Neuroscience, College of Veterinary Medicine, University of Florida, Gainesville, FL, 32611 USA; e Canadian Rivers Institute

**Keywords:** Environmental toxicology, Gene network, Neurotoxicity, Pesticide, Agrochemical

## Abstract

Fipronil is a phenylpyrazole pesticide that is used in both residential and agricultural applications. Fipronil is detected in run-off and water systems that are near areas in which the pesticide has been applied. The pesticide acts to antagonize gamma aminobutyric acid receptors, leading to over-excitation in the central nervous system. Fipronil has relatively high toxicity to fish, but the mechanisms underlying the toxicity are not well understood in embryonic stages. Zebrafish embryos were exposed to a single concentration of fipronil for 48 h at ∼3-4 h-post-fertilization. Following a 7-day depuration phase, transcriptome and behavioral analyses were conducted. Transcriptomics identified neural processes as those differentially expressed with different doses of fipronil (0.2 µg, 200 µg and 2 mg fipronil/L). Gene networks associated with astrocyte differentiation, myelination, neural tube development, brain stem response, innervation, nerve regeneration, astrocyte differentiation, among other pathways were altered with exposure. In addition, miRNA-related events are disrupted by fipronil exposure and genes associated with primary or pri-miRNA processing were increased in larval fish exposed to the pesticide. These data present putative mechanisms associated with neurological impacts at later ages of zebrafish. This is important because it is not clear how early exposure to pesticides like fipronil affect central nervous system function and organisms later in life.

## Specifications Table

**Table:** 

Subject	Biological Sciences, Omics: Transcriptomics
Specific subject area	Transcriptomics, pesticide, neurotoxicology, central nervous systems, mi-RNA
Type of data	Table Graph
How data were acquired	Microarray processing was performed according to manufacturer's protocols (Agilent Low RNA Input Fluorescent Linear Amplification Kit and Agilent 60-mer oligo microarray processing protocol, Agilent). The Agilent Zebrafish platform (V3, Catalog ID: G2519F-02647, Agilent) was used to probe samples. Microarray slides were scanned by Agilent DNA Microarray Scanner. Raw expression data along with tiff images were extracted by Agilent Feature Extraction Software (v10.7.3.1) which was used to extract spot intensity.
Data format	Raw Filtered Analyzed
Parameters for data collection	Fish were exposed to fipronil for 48 hours, and then allowed to depurate for 7 days in clean embyro rearing media. After 7 days, fish were pooled and microarrays were conducted on 9 dpf pools of larvae (3 fish per tube).
Description of data collection	Microarrays were performed in pools of zebrafish. Embryos were exposed for 48 h to one of the three doses of fipronil: Ethanol control (n = 6), low (0.2 µg/L) (n = 5), medium (200 µg/L) (n = 6) and high (2000 µg/L) (n = 6). RNA was extracted with TRIzol® for gene expression analysis. The supplemental data provides the gene abbreviations for fig. 2 and 3 and the connectivity (literature connection strength in the network) and probe value (fold change relative to control).
Data accessibility	‘With the article’ and on a public repository. Data are deposited in GEO Series GSE99608. The link is: https://www.ncbi.nlm.nih.gov/geo/query/acc.cgi?acc=GSE99608
Related research article	A.Eadie, I.C. Vásquez, X. Liang, X. Wang, C.L. Souders II, J. El Chehouri, R. Hoskot, A. Feswick, A.M. Cowie, J.R. Loughery, C.J. Martyniuk. 2020. Residual molecular and behavioral impacts of the phenylpyrazole pesticide fipronil in larval zebrafish (*Danio rerio*) following embryonic exposure. Comp. Biochem. Physiol. Part D: Genomics and Proteomics. In press [1]. https://doi.org/10.1016/j.cbd.2020.100743">

## Value of the Data

• Data are useful as they reveal molecular biomarkers of fipronil exposure
• Environmental risk assessment scientists, governments, and academics can use these data to develop environmental policies for water protection
• Data can be used or re-used to identify gene network biomarkers for pesticides in general, and can contribute to meta-analyses focused on biological responses to pesticide exposure
• Resource for which to compare other pesticides in terms of mechanism of action
• Society can benefit from understanding into how pesticide exposures affect wildlife and human health

## 1 Data Description

Fipronil is a phenylpyrazole agricultural pesticide that inhibits γ-amino-butyric acid (GABA_A_) receptors. Fipronil can be detected in aquatic ecosystems in the ng/L-µg/L range. In this study, we investigated whether an acute, environmentally relevant pulse exposure to fipronil during embryogenesis resulted in any lasting effects in larval zebrafish following a depuration period. Transcriptome profiling was conducted on 9 dpf larvae exposed to 0.2 µg fipronil/L (environmentally relevant), 200 µg and 2 mg fipronil/L for 48 h (∼4–52 hpf). A short pulse exposure to environmentally relevant levels of fipronil (first 2 days of development) was conducted.

**Fig. 1:**
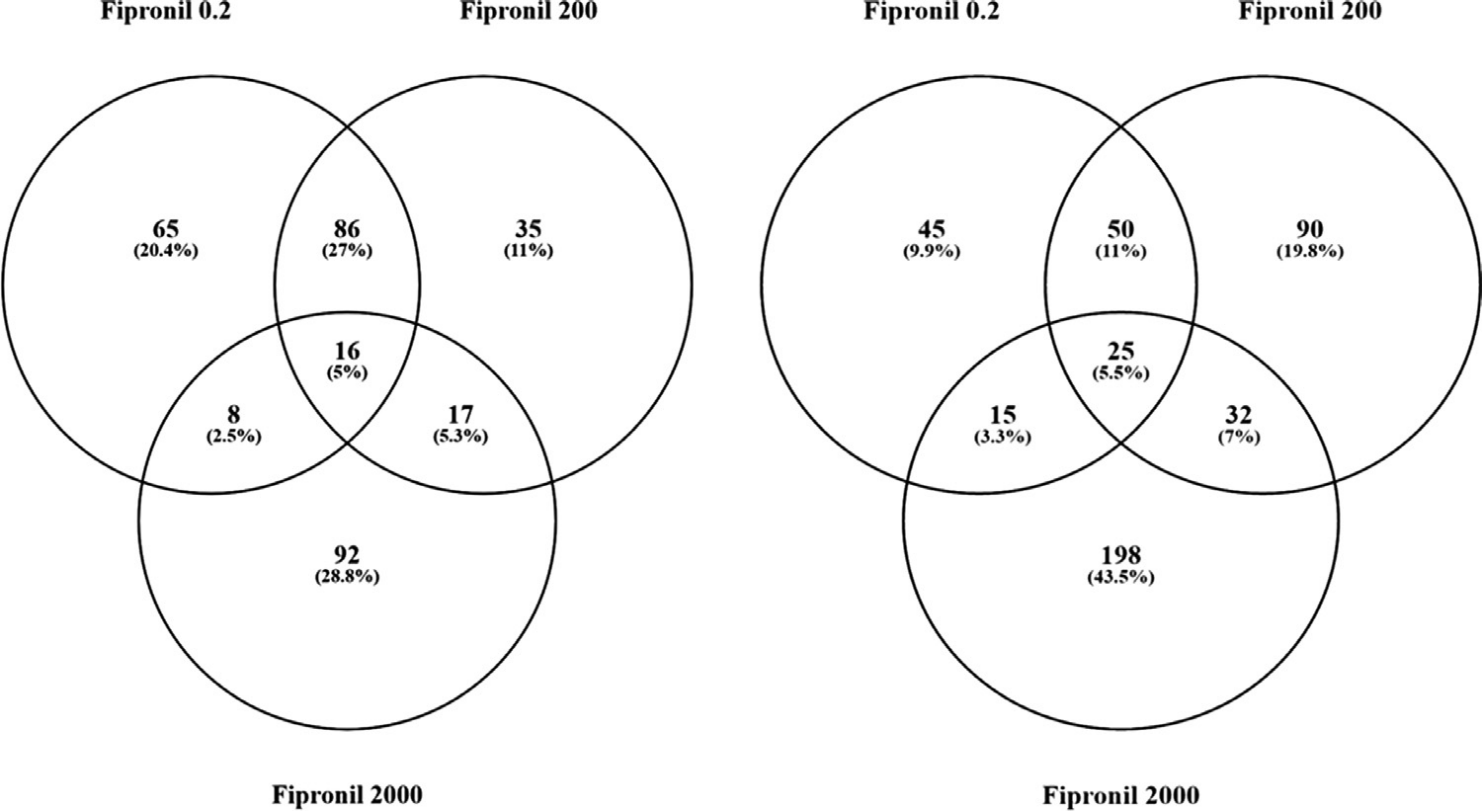
Venn diagrams for the number of gene sets (GSEA, left graph) and subnetworks (SNEA, right graph) affected in fish exposed to each of the three concentrations of fipronil. These computational methods represent two separate bioinformatics approaches used to discern molecular pathways. Units of concentrations are in µg/L.

**Fig. 2:**
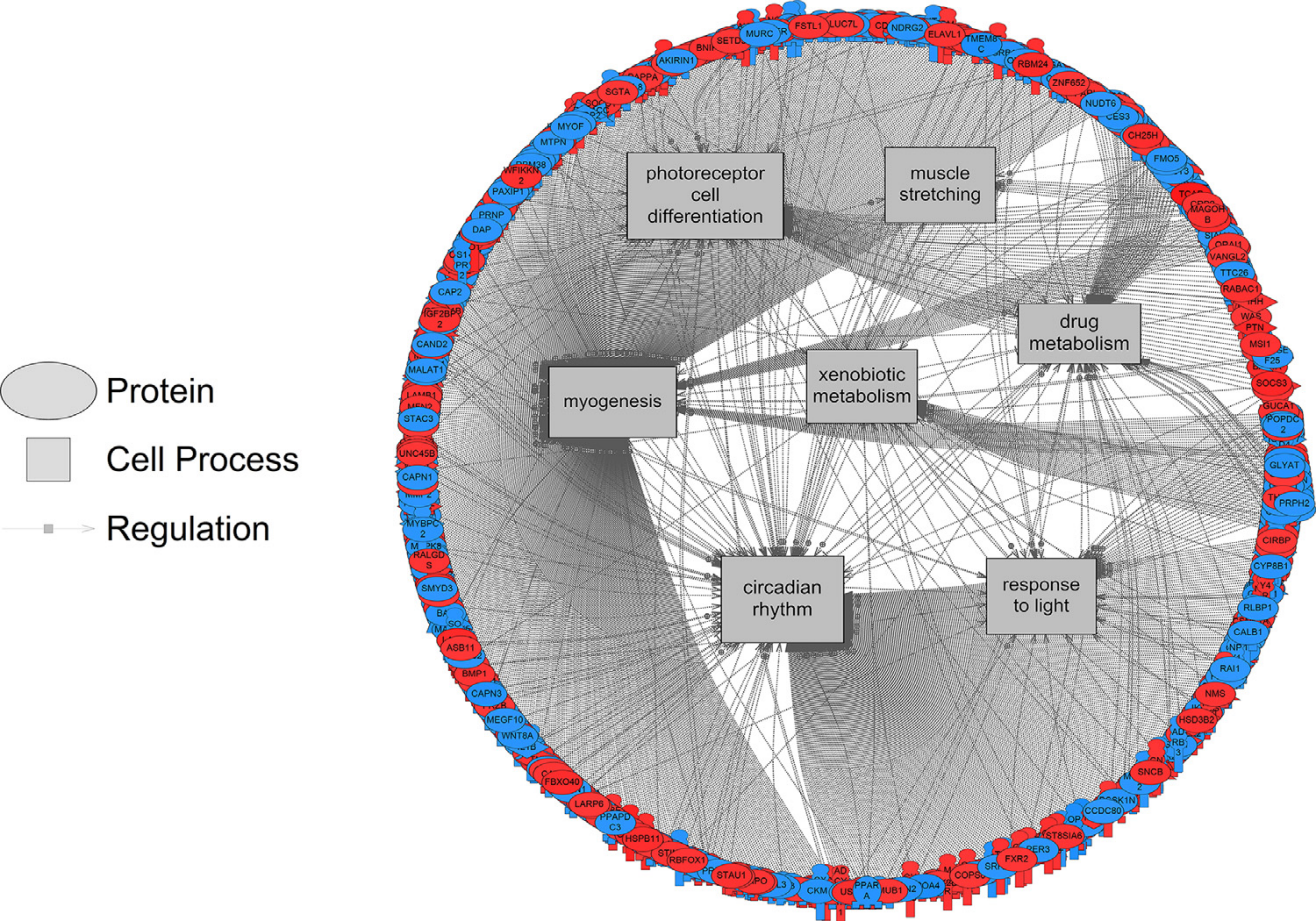
Gene network for cell processes affected in larval fish by fipronil exposure. Red indicates that the gene is increased in expression relative to the control and blue indicates a down-regulation. Data from the 200 µg fipronil/L exposure were used to build the network. Abbreviations are reported in Supplemental Data 1.

**Fig. 3:**
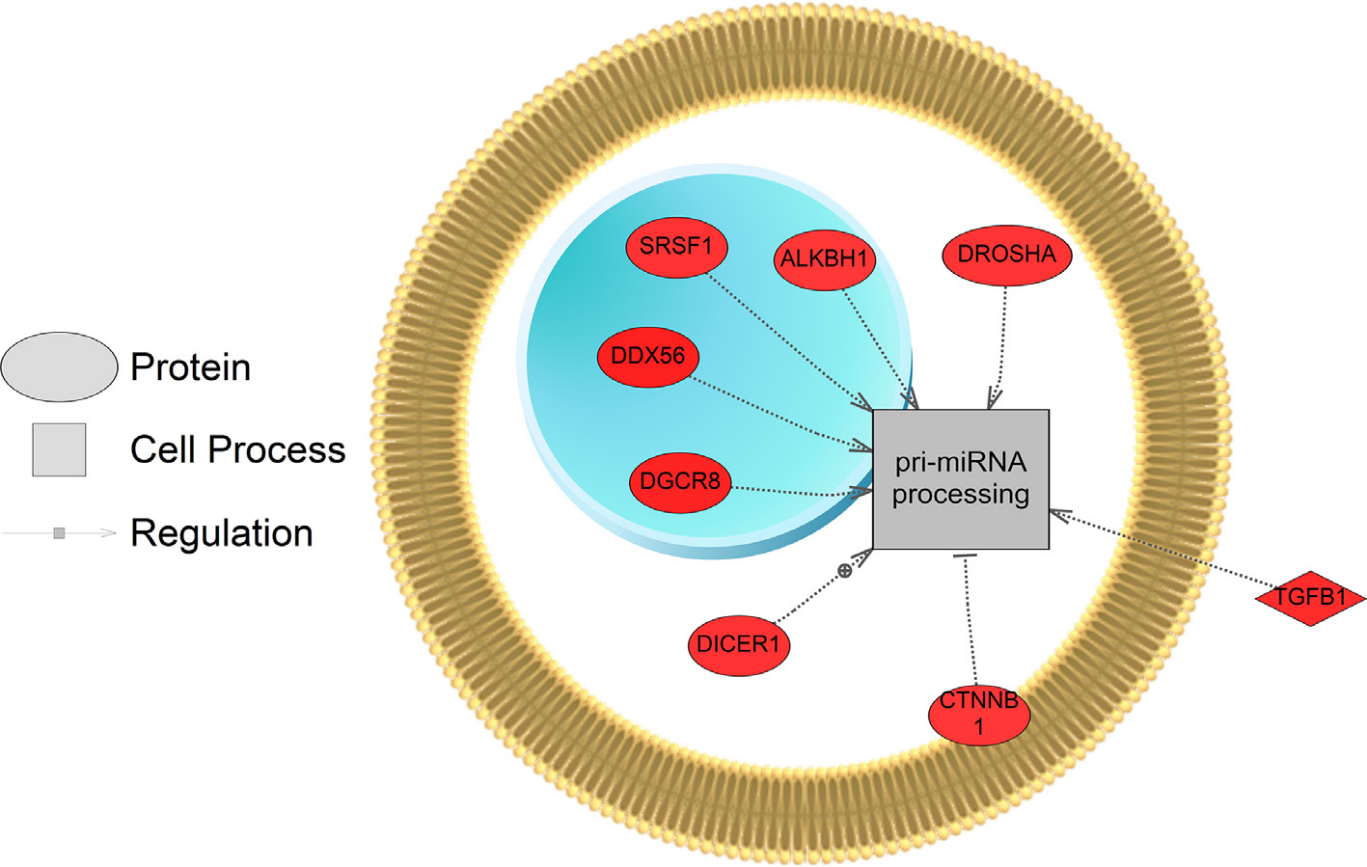
Gene network for a primary or pri-miRNA network affected in larval fish by fipronil exposure. Red indicates that the gene is increased in expression relative to the control and blue indicates a down-regulation. Data from the 200 µg fipronil/L exposure were used to build the network. Abbreviations are reported in Supplemental Data 1.

All results of the computational data analysis (section 2.3 below) can be found in the companion article as Supplemental Data 2 [1]. Here, these data were compared using a Venn Diagram (Oliveros, JC. (2007–2015)) [2]. Zebrafish exposed to the two lowest concentrations of the pesticide shared more gene sets in common (86 + 16 = 102) compared to fish exposed to the highest dose (2000 µg/L fipronil) (Fig. 1, left panel). These data are a concentration comparison for gene sets (GSEA) identified in the analysis. A similar result was observed for sub-network enrichment analysis and the two lower concentrations of fipronil shared 50 + 25 = 75 networks (Fig. 1, right panel). Eadie et al. [1] provides additional descriptions of the networks related to muscles and circadian rhythm. Also identified in the analysis were a significant number of networks associated with neurons and glial cells (Table 1). Larval fish exposed to the 0.2 µg/L concentration had 17 networks related to the central nervous system affected by fipronil, with many being downregulated. These included glial cell reaction, myelin maintenance, dendritic spine development, nervous system physiology, gliogenesis, glia cell migration, and astrocyte migration. Larval fish exposed to the 200 µg/L concentration had 6 networks related to the central nervous system affected by fipronil. These included astrocyte differentiation, myelination, neural tube development, brain stem response and astrocyte migration. Larval fish exposed to the 2000 µg/L concentration had 37 networks related to the central nervous system, nerve cell membrane potential, neurite development, nerve potential, generation of action potential, neuroendocrine cell differentiation, hypothalamus function, neurosecretion, neurotransmitter uptake, and brain maturation (Table 1). Fig. 2 depicts a combined network to highlight major processes affected by fipronil in the larval zebrafish after depuration. These processes included xenobiotic metabolism, myogenesis, drug metabolism, circadian rhythm, and response to light among others. Fig. 3 presents a gene network for primary or pri-miRNA. These are processes altered by fipronil that persist over a 7-day depuration phase. The supplemental data provides the gene abbreviations for Fig. 2 and 3 and the connectivity (literature connection strength in the network) and probe value (fold change relative to control).

**Table 1:** Sub-networks related to neural processes that were identified as significantly altered by fipronil at each concentration. Provided are the gene set seed, total number of neighbors in the network, number of measured neighbors, median fold change, and *p*-value.

Dose	Gene Set Seed	Total # of Neighbors	# of Measured Neighbors	Median change	p-value
0.2 µg/L	glial cell reaction	34	29	-1.15	0.019
	myelin maintenance	46	33	-1.15	0.011
	dendritic spine development	66	55	-1.10	0.014
	nervous system physiology	283	219	-1.09	0.043
	gliogenesis	157	126	-1.07	0.001
	glia cell migration	25	21	-1.07	0.033
	astrocyte migration	87	66	-1.06	0.010
	developmental process	695	567	-1.06	0.022
	synaptogenesis	787	596	-1.06	0.049
	myelination	468	385	-1.05	0.020
	astrocyte differentiation	115	92	-1.05	0.001
	dendrite morphogenesis	156	123	-1.04	0.005
	neural tube development	76	60	-1.03	0.006
	synaptic vesicle endocytosis	51	38	1.01	0.010
	nerve development	188	155	1.03	0.015
	Schwann cell formation	25	23	1.05	0.020
	dendritic extension	27	21	1.26	0.003

200 µg/L	astrocyte differentiation	115	92	-1.06	0.005
	myelination	468	385	-1.03	0.012
	neural tube development	76	60	1.05	0.021
	brain stem response	10	10	1.11	0.035
	astrocyte migration	87	66	1.12	0.038
	Schwann cell formation	25	23	1.14	0.003

2000 µg/L	ganglion stimulation	5	5	-1.21	0.019
	hippocampus rhythm	17	11	-1.17	0.037
	neurocognition	21	16	-1.15	0.007
	gliogenesis	157	126	-1.10	0.004
	brainstem development	11	10	-1.09	0.013
	glia proliferation	71	56	-1.07	0.002
	cerebellum development	80	70	-1.07	0.023
	nervous system physiology	283	219	-1.05	0.003
	neural precursor cell proliferation	230	188	-1.05	0.023
	myelination	468	385	-1.04	0.006
	nerve maturation	36	27	-1.03	0.006
	neurogenesis	1129	886	-1.02	0.043
	cerebral cortex development	39	34	1.01	0.026
	hippocampal function	158	116	1.03	0.043
	axon guidance	346	289	1.04	0.035
	brain stem response	10	10	1.05	0.032
	innervation	323	263	1.05	0.005
	nerve regeneration	319	256	1.05	0.012
	astrocyte differentiation	115	92	1.06	0.039
	hypothalamus development	11	8	1.06	0.014
	transmission of nerve impulse	645	467	1.06	0.033
	synaptogenesis	787	597	1.06	0.041
	dopaminergic system	157	117	1.07	0.026
	peripheral nerve excitation	39	31	1.07	0.010
	hippocampus plasticity	98	82	1.07	0.026
	central nervous system function	163	123	1.07	0.025
	neuromuscular synaptic transmission	66	53	1.07	0.017
	remyelinization	167	135	1.07	0.015
	nerve cell membrane potential	34	25	1.07	0.026
	neurite development	62	49	1.08	0.036
	nerve potential	62	44	1.09	0.022
	generation of action potential	127	89	1.14	0.002
	neuroendocrine cell differentiation	17	13	1.14	0.015
	hypothalamus function	85	65	1.15	0.014
	neurosecretion	64	52	1.17	0.010
	neurotransmitter uptake	26	22	1.23	0.013
	brain maturation	36	32	1.26	0.021

## 2 Experimental Design, Materials and Methods

### 2.1 Experimental design

Microarray analysis was performed on whole larvae exposed to three doses of fipronil: Ethanol control (n = 6), low (0.2 μg/L) (n = 5), medium (200 μg/L) (n = 6) and high (2000 μg/L) (n = 6). Fish were exposed to a short-term pulse of fipronil (48 h exposure) at environmentally relevant concentrations. Each experimental dose was performed in quadruplicate and solutions were renewed after 24 h of incubation. Fish were placed into an Incubating Microplate Shaker (VWR International; Mississauga, Ontario) at 27 ± 1°C and 100 rpm. After 48 h of treatment, a subset of individuals was transferred to clean water for an addition 7 days.

### 2.2 Microarray analysis and scanning

Following depuration, RNA was extracted from the larvae (n = 3 / pool) using the Qiagen RNeasy® Mini Kit (Qiagen) as per the manufacturer's protocol. The Nanodrop 2000 Spectrophotometer (Thermo Scientific, Wilmington, DE, USA) and 2100 Bioanalyzer (Agilent, Santa Clara, CA, USA) were used to assess the quality of the RNA samples. Microarrays were processed as per [3,4]. Raw expression data along with tiff images were extracted by Agilent Feature Extraction Software (v10.7.3.1). All microarray data adhered to established guidelines “Minimum Information About a Microarray Experiment (MIAME)” (http://www.ncbi.nlm.nih.gov/geo/info/MIAME).

Differentially expressed genes (DEGs) were identified following normalization of all microarrays using Quantile Normalization. The arrays were quality control checked using a distribution analysis that plots the intensity distributions of each microarray slide to ensure these distributions are relatively equal. To reduce noise, normalized intensity data were filtered based on the limit of detection of the microarrays (2.1 intensity) based on control probes and standard curve form the “RNA Spike-In mix”. A one-way ANOVA, followed by an FDR correction (*p* < 0.05) for multiple tests using the non-permutation based Benjamini and Hochberg method identified genes affected by fipronil in larvae. All expression data were deposited into Gene Expression Omnibus, an open source repository for transcriptomics data (GSE99608).

### 2.3 Bioinformatics and network analysis

Gene network analysis using Pathway Studio (v11) (Elsevier) was conducted with the Name + Alias feature. Each fipronil dose was analyzed separately for gene set enrichment which employed 1000 permutations of fold change data using a Kolmogorov–Smirnov test. The computational pipeline can be found in Martyniuk et al., [4]. The enrichment *p*-value for all queries was set at *p* < 0.05. All networks are provided in Supplemental Data 1.

Supplemental Data 1. Abbreviations for figures.

## Ethics Statement

All experiments adhered to ethics policies for the University of New Brunswick Saint John and the University of Florida. Experimental procedures were approved by Institutional Animal Care and Use Committee (IACUC) at both UNB and UF (IACUC Study #201708562).

## CRediT Author Statement

**Ashley Eadie:** experimentation, manuscript writing; **Isabel Cristina Vásquez:** experimentation; Xuefang Liang: experimentation; **Xiaohong Wang:** experimentation; **Christopher L. Souders II:** supervision experimentation; **Jana El Chehouri:** experimentation; **Rohit Hoskote:** experimentation; **April Feswick:** supervision, experimentation; **Andrew M. Cowie:** supervision, experimentation; **Jennifer R. Loughery:** supervision, experimentation; **Christopher J. Martyniuk:** supervision, manuscript preparation, data analysis.

## Declaration of Competing Interest

The authors declare that they have no known competing financial interests or personal relationships which have or could be perceived to have influenced the work reported in this article.

## Appendix Supplementary materials

Image, application 1

## Footnotes

Supplementary material associated with this article can be found in the online version at doi:10.1016/j.dib.2020.106413.
